# Gut Microbiome Signatures Across Migratory, Sedentary, and Aquaculture Ecotypes of *Coilia nasus*

**DOI:** 10.3390/ani16050840

**Published:** 2026-03-07

**Authors:** Xue Liu, Congping Ying, Fengjiao Ma, Yanping Yang, Kai Liu

**Affiliations:** 1 Wuxi Fisheries College, Nanjing Agricultural University, Wuxi 214082, China; 2023113008@stu.njau.edu.cn; 2 Key Laboratory of Freshwater Fisheries and Germplasm Resources Utilization, Ministry of Agriculture and Rural Affairs, Freshwater Fisheries Research Center, Chinese Academy of Fishery Sciences, Wuxi 214082, China; yingcongping@ffrc.cn (C.Y.); mafengjiao2019@163.com (F.M.); yangyp@ffrc.cn (Y.Y.)

**Keywords:** *Coilia nasus*, intestinal microbiota, 16S rRNA sequencing, ecotype

## Abstract

*Coilia nasus*, a typical species with migratory–sedentary polymorphism, shows different intestinal microbiota characteristics among its different ecotypes. This is attributed to differences in feeding habits and habitat environments (such as water temperature, salinity, etc.). This study constructed a database of intestinal microbiota for three ecological types of *C. nasus*, namely migratory type (comprising marine populations and freshwater populations), sedentary type and aquaculture-reared type, through 16S rRNA amplicon sequencing technology. This study investigates the ecological mechanisms underlying microbiota differentiation, focusing on three key drivers: environmental selection, host nutritional metabolism requirements, and host life history strategies. The results showed that the core flora of *C. nasus* consisted of Firmicutes, Proteobacteria, and Actinobacteria. Both the depletion of microbial taxa and the enrichment of marine-adapted bacterial lineages—including Proteobacteria and Psychrobacter—are associated with elevated salinity in the migratory marine population of *C. nasus*. In contrast, the elevated relative abundance of Actinobacteria in aquaculture-reared *C. nasus* is likely attributable to dietary supplementation with protein- and lipid-rich artificial feed. Functional correlation analysis holds promise for partially predicting the microbiota’s metabolic functional succession patterns. The dominance of *Pseudomonas_E* in the migratory freshwater population is consistent with its well-documented physiological versatility and adaptive capacity in dynamically fluctuating aquatic habitats. The elevated abundance of Cyanobacteria in the sedentary population *C. nasus* coincides with the water bloom in their habitat, suggesting that the structure of the microbiota may serve as a novel biomarker for indicating the ecosystem. In conclusion, this study identifies potential molecular markers for tracing genetic resources and distinguishing ecological types of *C. nasus*, while establishing a theoretical foundation for elucidating the co-evolutionary dynamics between fish hosts and their associated microbiota—and thereby informing both conservation strategies for wild populations and microbiota-informed aquaculture practices.

## Simple Summary

This study constructed a database of intestinal microbiota for three ecological types of *Coilia nasus*, namely migratory type (comprising marine population and freshwater population), sedentary type and aquaculture-reared type, through 16S rRNA amplicon sequencing technology. This study investigates the ecological mechanisms underlying microbiota differentiation, focusing on three key drivers: environmental selection, host nutritional metabolism requirements, and host life history strategies. The results showed that the core flora of *Coilia nasus* consisted of Firmicutes, Proteobacteria, and Actinobacteria. Both the depletion of microbial taxa and the enrichment of marine-adapted bacterial lineages—including Proteobacteria and Psychrobacter—may be associated with elevated salinity in the migratory marine population of *Coilia nasus.* Significant variations in both richness and diversity of the intestinal microbiota were observed among the different ecological groups. In conclusion, this study not only provides potential molecular markers for the traceability of the genetic resources and the identification of ecological types of *Coilia nasus*, but also lays a theoretical foundation for understanding the co-evolutionary mechanism between fish hosts and microorganisms and for formulating conservation strategies for wild populations and aquaculture strategies based on microbiota regulation.

## 1. Introduction

*Coilia nasus* (hereinafter referred to as *C. nasus*), belonging to the Engraulidae family within the Clupeiformes order, exhibits unique ecological adaptability that makes it an important model for studying fish resource conservation [1]. This species demonstrates two distinct ecological phenotypes: anadromous migration and freshwater residency. The former is extensively distributed across estuarine ecosystems along the northwest Pacific coast. During spring, mature individuals of this population exhibit a reproductive-driven bidirectional migration pattern between marine and freshwater environments, thereby developing a unique adaptive capability to salinity gradients [2]. Conversely, the latter primarily resides in lake ecosystems in the middle and lower reaches of the Yangtze River (e.g., Taihu Lake and Chaohu Lake), playing a crucial role in the regional biotic community by occupying a key ecological niche [3,4]. *C. nasus* has been classified as Endangered (EN) on the IUCN Red List of Threatened Species (www.iucnredlist.org, accessed on 25 February 2026), underscoring the critical and immediate need for conservation action.

In recent years, research into the composition, structure, and functional dynamics of host-intestinal microbiota interactions has advanced rapidly. The intestinal microbiome of fish serves as the “second genome” in host-environment interactions and plays a critical role in shaping the host’s nutritional metabolism and health homeostasis via pathways such as metabolic regulation and immune modulation [5,6,7,8]. Research demonstrates that the species-specific structure of the intestinal microbiota is collectively shaped by a variety of factors, including the host’s ecological lifestyle (migratory versus sedentary), geographical population distribution, developmental stages, and feeding strategies [9,10,11,12]. Compared with terrestrial vertebrates, the intestinal microbiota of fish exhibits pronounced dynamic variations and relatively lower biodiversity [13]. However, in widely distributed carnivorous species, discernible core microbiota are still present. These conserved microbial groups may harbor critical information regarding the coevolutionary processes between hosts and their associated microbiota [14,15,16,17].

Current research on *C. nasus* is predominantly concentrated in the Yangtze River estuary, its middle and lower reaches, and selected tributaries and connected lakes. Key research themes encompass population genetics [18]; age composition and somatic growth dynamics [19]; geographic population differentiation and classification [20,21]; stock assessment and demographic structure [22,23]; and integrative genomics—including functional annotation of differentially expressed genes and pathway enrichment analyses [24,25,26]. In contrast, investigations into the intestinal microbiota of *C. nasus* remain comparatively limited, with existing studies largely confined to intraspecific comparisons within a single ecotype (e.g., anadromous or freshwater-resident forms) [27,28]. Notably, a systematic, cross-ecotype comparative analysis of gut microbial community composition, diversity, and functional potential across distinct ecological groups of *C. nasus* is still lacking.

In recent years, driven by the rapid advancement of high-throughput sequencing technologies, 16S rRNA amplicon sequencing [29] has enabled high-resolution profiling of the intestinal microbiota. This study collected intestinal samples from three ecologically distinct populations of *C. nasus*: anadromous individuals (comprising both marine-phase and freshwater-phase migrants), sedentary individuals, and aquaculture-reared individuals. The primary objective was to establish a more comprehensive, resolved catalog of bacterial taxa inhabiting the gut microbiota of *C. nasus*. The study introduces three key conceptual and methodological advances: (1) the first systematic, multi-ecotype comparison of gut microbiota composition and predicted functional profiles across life history-divergent forms of *C. nasus*; (2) comparing the convergent and ecotype-specific patterns in gut microbiota structure across the anadromous, sedentary, and aquaculture-reared ecotypes of *C. nasus*; and (3) an integrative investigation linking inter-ecotype differences in microbiome structure and function to host-associated environment and dietary regimes, thereby advancing mechanistic understanding beyond mere correlation. These findings provide a novel theoretical framework for understanding host–microbe symbiosis in fish, while also offering a microbial ecological basis for formulating conservation and aquaculture strategies for *C. nasus* populations.

## 2. Materials and Methods

### 2.1. Sampling of C. nasus and Their Intestinal Tissues

**Figure 1:**
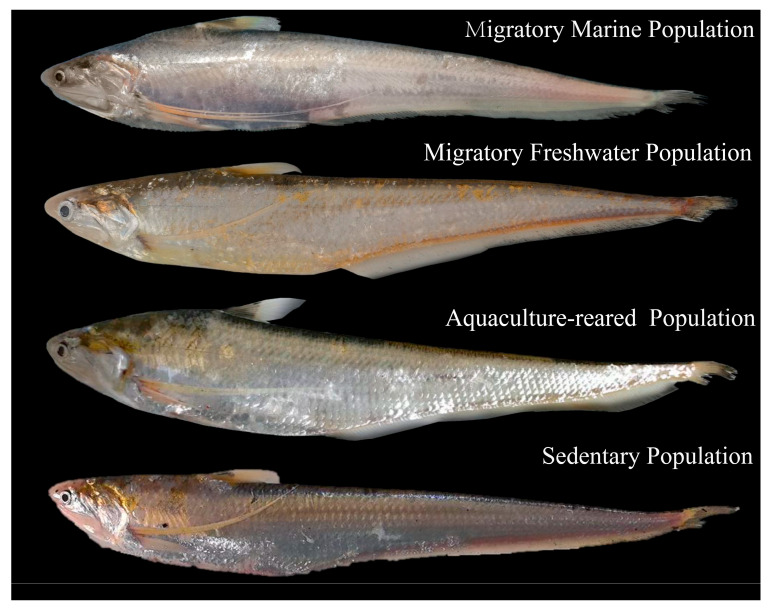
Morphological illustrations of different ecological populations of *C. nasus*.

**Figure 2:**
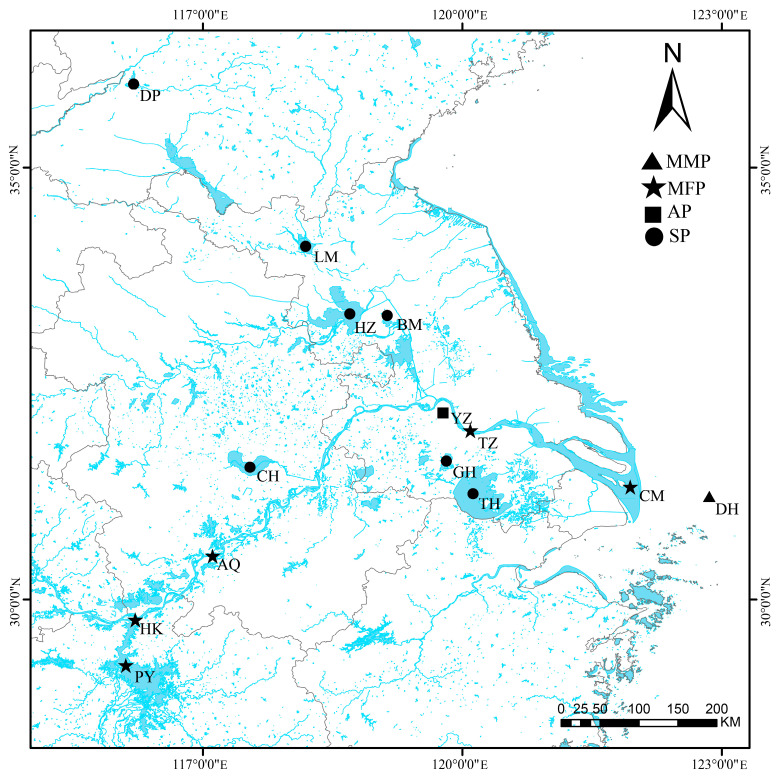
Sampling locations of different ecological populations of *C. nasus*. Among these, the migratory marine population of *C. nasus* were collected outside the no-fishing zone in the East China Sea (DH). The migratory freshwater population of *C. nasus* were sampled from four locations along the Yangtze River, including Chongming Island (CM), Taizhou (TZ), Anqing (AQ), and Hukou (HK), as well as from Poyang Lake (PY). The aquaculture-reared population of *C. nasus* was obtained from the Yangzhong aquaculture base (YZ). The sedentary population of *C. nasus* were collected from Dongping Lake (DP), Luoma Lake (LM), Hongze Lake (HZ), Baima Lake (BM), Geyang Lake (GH), Taihu Lake (TH) and Chaohu Lake (CH).

To ensure comprehensive coverage of *C. nasus* sample types, our experimental group collected migratory marine population (MMP) of *C. nasus* from the East China Sea, migratory freshwater population (MFP) of *C. nasus* from the main stream of the Yangtze River and Poyang Lake, aquaculture-reared population (AP) of *C. nasus* from Yangzhong aquaculture bases and sedentary population (SP) of *C. nasus* from landlocked lakes. Figure 1 presents morphological illustrations of different ecological populations of *C. nasus* and Figure 2 illustrates sampling locations of different ecological populations of *C. nasus*. All individuals were confirmed as migratory *C. nasus* based on the Sr/Ca ratio analysis of otoliths (Figure S1). At each sampling site, 20 individuals of *C. nasus* were collected; morphometric and biological data were recorded for each individual. A total of 280 individuals were sampled across all ecological groups. Group-level statistics, including mean ± SD of total length and body weight, were then calculated separately for the anadromous, aquaculture-reared, and sedentary ecological populations (Table 1).

After measuring the biological indicators, *C. nasus* were immediately dissected on site. The external surface of each was sterilized with an ethanol-soaked cotton ball. Subsequently, the abdomen was carefully incised from the anus upward along the ventral ridge using scissors. The entire intestine was then isolated and transferred to a 2 mL cryotube. The samples were rapidly frozen in liquid nitrogen and subsequently stored at −80 °C in an ultra-low temperature freezer.

### 2.2. Extraction and Sequencing of Total Bacterial Genomic DNA

Total DNA from the intestinal microbiota was extracted using the E.Z.N.A.^®^ Soil DNA Kit (Omega Bio-Tek, Norcross, GA, USA, Cat. No. D5624-02). The quality of DNA was assessed by 1% agarose gel electrophoresis. The concentration and purity of DNA were measured using a NanoDrop 2000 spectrophotometer (Thermo Fisher Scientific, Waltham, MA, USA). The V3–V4 variable region was amplified using the upstream primer (5′-CCTAYGGGRBGCASCAG-3′) and the downstream primer (5′-GGACTACNNGGGTATCTAAT-3′) [30]. Following PCR amplification, sequencing was conducted on the Illumina MiSeq platform (Illumina, San Diego, CA, USA), with three technical replicates established for consistency.

The PCR components involved Q5 reaction buffer (5×, 5 μL), Q5 High-Fidelity GC buffer (5×, 5 μL), Q5 High-Fidelity DNA Polymerase (5U/μL, 0.25 μL), each Forward and Reverse primer (10 μM, 1 μL), dNTPs (10 mM, 2 μL), DNA Template (2 μL), and ddH_2_O (8.75 μL). Thermal cycling consisted of initial denaturation at 98 °C for 5 min, followed by 25 cycles (denaturation at 98 °C for 30 s, annealing at 52 °C for 30 s, and extension at 72 °C for 45 s) with a final extension of 5 min at 72 °C. A total of PCR amplicons were purified with Agencourt AMPure Beads (Beckman Coulter, Indianapolis, IN, USA) and quantified using the PicoGreen dsDNA Assay Kit (Invitrogen, Carlsbad, CA, USA). After the individual quantification step, amplicons were pooled in equal amounts, and Single Molecule Real Time (SMRT) sequencing technology was performed using the PacBio Sequel platform at Shanghai Personal Biotechnology Co., Ltd. (Shanghai, China).

### 2.3. Bioinformatics Analysis of High-Throughput Sequencing Data

The primer fragments were removed from the sequences using Qiime’s cutadapt trim-paired, and sequences that did not match the primers were discarded. Subsequently, DADA2 was employed for sequence quality control, denoising, merging, and chimera removal. DADA2-based quality filtering was performed with the following parameters: truncLen = c(240, 200), maxN = 0, maxEE = c(2, 2), and truncQ = 2. Putative chimeric sequences were removed using the removeBimeraDenovo function (default settings). ASVs were taxonomically assigned to the species level using the greengenes2 reference database, which integrates phylogenetic frameworks from the Genome Taxonomy Database (GTDB) and refines broad bacterial taxonomic groups—such as the deeply branching lineages—into more granular, phylogenetically informed categories. After denoising, the feature sequences and ASV tables of ASVs (Amplicon Sequence Variants) were merged, and Singleton ASVs (ASVs with a total sequence count of only 1 across all samples) were removed. In the raw data obtained from high-throughput sequencing, an R script was used to analyze the length distribution of high-quality sequences in all samples, followed by ASV analysis on the final sequences.

Further analysis of species composition among samples was performed based on bacterial ASVs identified in the intestines. In this study, the relative abundance of each bacterium at a specific taxonomic level was visualized using bar charts, with particular attention given to the core microbiota of *C. nasus*. Astudillo-Garcia et al. proposed that defining the common core bacterial group (i.e., the bacterial community within the microbiome) requires setting both a detection threshold (relative abundance) and an existence rate percentage for bacterial classifications [31]. The range of common core annotations spans from as low as 30% [32] to 100% occurrence rates [33], while the detection threshold varies between 0.001% and 0.1%. In our study, bacterial ASVs present in over 50% of the same sample group were designated as part of the core microbiota (the core microbiota was defined as bacterial ASVs detected in at least 10 of the 20 individuals sampled from the same location).

A Venn diagram was employed to illustrate shared and unique bacterial species and their respective counts in the intestines of different *C. nasus* groups, and the contribution of each group’s intestinal microbiota to the total bacterial community was calculated (the contribution rate was calculated as the number of intestinal bacteria in each group of *C. nasus* divided by the total number of bacteria). The LEfSe method was utilized to identify robust differences in species between groups. The functional potential of the microbial communities was predicted using PICRUSt2 (v2.5.2) with the MetaCyc pathway database (v12.5) as the reference annotation resource.

## 3. Results and Analysis

### 3.1. Construction of the Background Database for Intestinal Microbiota in C. nasus

A total of 23,764,710 high-quality 16S rRNA gene sequences were obtained from intestinal samples of 280 individuals of *C. nasus*, with read lengths ranging from 235 to 446 base pairs (bp). These sequences were clustered into 33,371 prokaryotic amplicon sequence variants (ASVs). The dilution curves demonstrated that each curve became increasingly flat as the number of sequences increased, suggesting that the sequencing depth was adequate to capture the information of all microorganisms present in the samples (Figure S2). A total of 37 phyla, 106 classes, 280 orders, 525 families, 1144 genera, and 1707 species were identified in the intestinal samples of *C. nasus*. After averaging across the samples, the dominant bacterial taxa were statistically analyzed based on their relative abundances (Table S1). Among these, the top four dominant phyla collectively accounted for 92.20% of the total abundance, specifically Firmicutes, Proteobacteria, Actinobacteriota, and Cyanobacteria. In contrast, the top five dominant genera accounted for only 39.90% of the total abundance, namely *Clostridium_T*, *Plesiomonas*, *Unclassified_f_Peptostreptococcaceae*, *Clostridium_P*, and *Pseudomonas_E*.

### 3.2. Alpha and Beta Diversity Analysis of the Intestinal Microbiota of C. nasus

**Figure 3:**
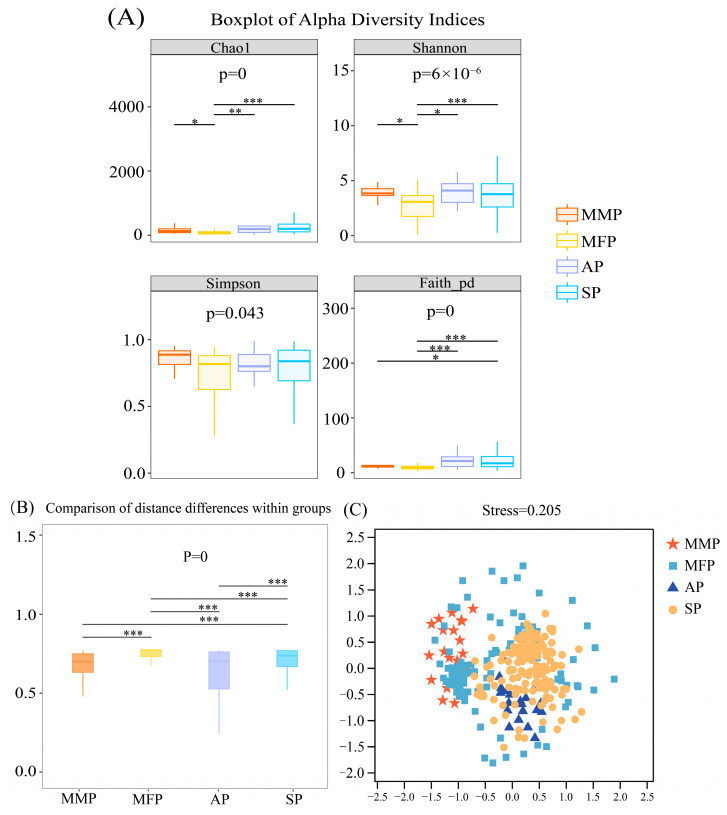
Alpha and beta diversity analysis of the intestinal microbiota of *C. nasus*. (**A**) Boxplot of alpha diversity indices, where asterisks (*) denote significant differences among ecological groups. * denotes significance, ** denotes high significance, and *** denotes extreme significance. A greater number of asterisks (*) indicates more pronounced inter-group differences. The acronyms are defined as follows: MMP, Migratory Marine Population; MFP, Migratory Freshwater Population; AP, Aquaculture-reared Population; and SP, Sedentary Population. (**B**) Inter-group beta diversity differences among the four ecological populations of *C. nasus* (MMP, MFP, AP, and SP) were assessed using the Kruskal–Wallis test, followed by Dunn’s post hoc test correction for pairwise comparisons (Table S3). (**C**) Beta diversity assessed via non-metric multidimensional scaling (NMDS), illustrating the distribution patterns and dissimilarities of microbial communities across samples.

Alpha diversity indices were compared across the four ecological populations of *C. nasus* (MMP, MFP, AP, and SP) using the Kruskal–Wallis test, followed by Dunn’s post hoc test with Benjamini–Hochberg false discovery rate (FDR) correction for pairwise comparisons. Specifically, the Chao1 index was used to estimate species richness, whereas the Shannon and Simpson indices were applied to evaluate community diversity (Figure 3A). Significant differences in both microbial richness and alpha diversity were observed across the four ecological populations of *C. nasus*, with the marine-migratory (MMP) and freshwater-migratory (MFP) populations exhibiting the greatest divergence in intestinal microbiota composition (*p* < 0.05). Notably, the aquaculture-reared population (AP) displayed significantly higher microbial diversity and richness compared to the other groups (Table S2).

Inter-group beta diversity difference analysis among the four ecological populations of *C. nasus* revealed significant pairwise differences among all group comparisons (*p* < 0.01), as indicated by asterisks (*) (Figure 3B). Beta diversity analysis was conducted to examine the structural composition of intestinal microbial communities across ecological types (Figure 3C). Non-metric multidimensional scaling (NMDS) was utilized to visualize community relationships, where shorter intergroup distances indicate greater similarity in microbial assemblages. The NMDS plot revealed clear separation among the four ecological groups, with only minor overlap, indicating distinct microbial community structures across groups. Among these, the migratory freshwater population (MFP) exhibited the highest degree of dispersion, followed by the sedentary population (SP), whereas the aquaculture-reared population (AP) showed the least variation, suggesting greater homogeneity in its intestinal microbiota.

### 3.3. The Structure of the Intestinal Microbiota in Different Ecological Populations of C. nasus

**Figure 4:**
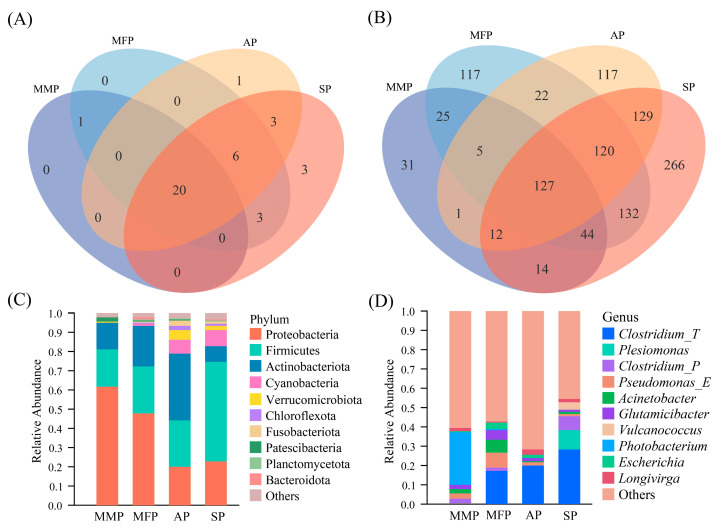
Composition and distribution of intestinal bacteria in four ecological groups of *C. nasus*. (**A**) Venn diagram showing bacterial distribution at the phylum level. The acronyms are defined as follows: MMP, Migratory Marine Population; MFP, Migratory Freshwater Population; AP, Aquaculture-reared Population; and SP, Sedentary Population. (**B**) Venn diagram illustrating bacterial distribution at the genus level. (**C**) Species composition of dominant bacterial phyla in the intestinal tract of *C. nasus* (relative abundance > 0.1%). (**D**) Species composition of dominant bacterial genera in the intestinal tract of *C. nasus* (relative abundance > 0.1%).

A petal diagram was employed to visualize community composition and identify group-specific species across the four ecological populations of *C. nasus*. At the phylum level (Figure 4A), the four ecological groups of *C. nasus* shared 20 bacterial phyla. The migratory marine population (MMP) was identified with 21 bacterial phyla, contributing 56.76% to the total bacterial phyla of *C. nasus*. The migratory freshwater population (MFP) was identified with 30 bacterial phyla, contributing 81.08%. Similarly, the aquaculture-reared population (AP) contained 30 bacterial phyla, with Elusimicrobiota being its unique phylum. The sedentary population (SP) was identified with 35 bacterial phyla, contributing as high as 94.59%, and Campylobacterota, WOR-3, and Desulfobacterota_E were exclusively present in this group. Similarly, at the genus level (Figure 4B), the four ecological groups of *C. nasus* shared 127 bacterial genera. Specifically, the MMP harbored 259 bacterial genera, contributing only 22.64% to the total bacterial genera, with 31 unique genera. The MFP contained 592 bacterial genera, contributing 51.75%. The AP had 533 bacterial genera, contributing 46.59%. Lastly, the SP harbored 844 bacterial genera, contributing 73.78% (Table S4).

To compare the differences in the relative abundance of intestinal microbiota among different ecological groups of *C. nasus*, this study tallied the top 10 species compositions at the phylum and genus levels, respectively. At the phylum level (Figure 4C), Proteobacteria, Firmicutes, and Actinobacteriota were the dominant bacterial phyla in *C. nasus*, accounting for 78.81%~94.99% of the total abundance. Notably, the relative abundance of Firmicutes in the intestinal microbiota of the SP was higher than that in other ecological types of *C. nasus*. At the genus level (Figure 4D), bacterial groups with lower abundance rankings accounted for a significantly larger proportion, contrasting with observations at the phylum level. Notably, *Photobacterium* exhibited much higher relative abundance in the intestinal microbiota of the MMP *C. nasus* compared to other ecological types. *Clostridium_T* showed relatively high abundance in the MFP, AP, and SP of *C. nasus*. Additionally, *Plesiomonas* was markedly more abundant in the sedentary group than in other ecological types of *C. nasus*.

### 3.4. The Intestinal Microbiota of Migratory C. nasus Varies Under Different Living Environments

**Figure 5:**
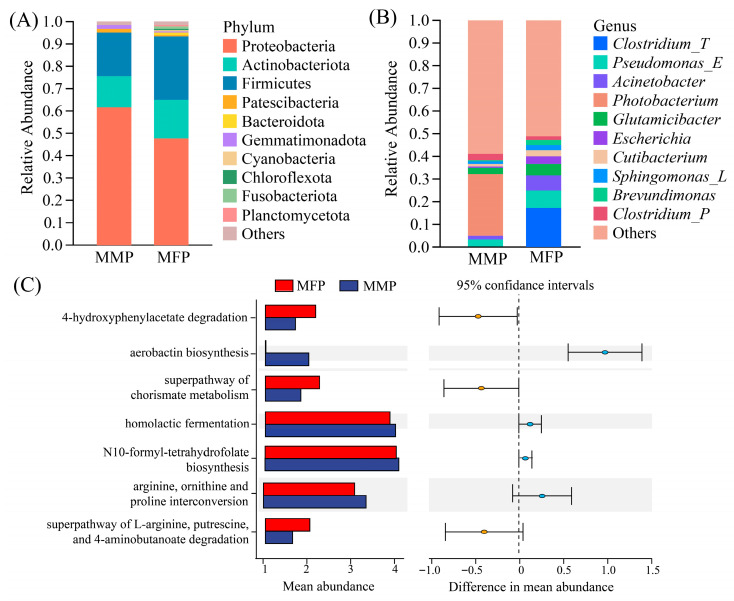
Statistical analysis of the intestinal microbiota composition and metabolic pathways in anadromous marine and freshwater populations of *C. nasus*. MMP, Migratory Marine Population, and MFP, Migratory Freshwater Population. (**A**) Composition of dominant bacterial phyla in the intestinal tract of *C. nasus* (relative abundance > 0.1%). (**B**) Composition of dominant bacterial genera in the intestinal tract of *C. nasus* (relative abundance > 0.1%). (**C**) Differential metabolic pathways in *C. nasus* (95% confidence interval).

The intestinal microbiota of anadromous *C. nasus*, which migrate from the ocean to the Yangtze River (freshwater environment) for reproductive migration, exhibit significant differences due to variations in external environmental factors, including salinity and chemical composition. From the perspective of species composition, at the phylum level (Figure 5A), the dominant bacterial phyla in both migratory marine (MMP) and freshwater populations (MFP) of *C. nasus* were Proteobacteria, Actinobacteriota and Firmicutes, collectively accounting for 93.46% to 94.99%. At the genus level (Figure 5B), the dominant bacterial genera in marine-migratory *C. nasus* were *Photobacterium*, *Psychrobacter*, and *Aliivibrio*, whereas those in the MFP *C. nasus* were *Clostridium_T*, *Pseudomonas_E*, and *Acinetobacter*.

Further analysis of the metabolic pathways exhibiting significant differences between the MMP and the MFP of *C. nasus* revealed that the MMP was enriched in aerobactin biosynthesis and arginine, ornithine, and proline interconversion pathways. While the MFP of *C. nasus* was enriched in the pathways of 4-hydroxyphenylacetate degradation, superpathway of chorismate metabolism, and superpathway of L-arginine, putrescine, and 4-aminobutanoate degradation (Figure 5C).

### 3.5. The Intestinal Microbiota Composition Differences Between Aquaculture-Reared and Sedentary Population C. nasus

**Figure 6:**
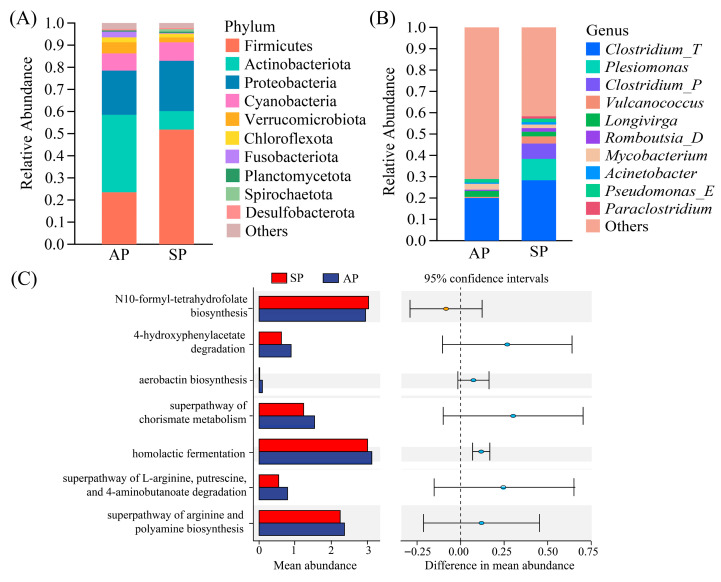
Statistical analysis of the intestinal microbiota composition and metabolic pathways in aquaculture-reared population and sedentary population *C. nasus*. AP, Aquaculture-reared Population and SP, Sedentary Population. (**A**) Composition of dominant bacterial phyla in the intestinal tract of *C. nasus* (relative abundance > 0.1%). (**B**) Composition of dominant bacterial genera in the intestinal tract of *C. nasus* (relative abundance > 0.1%). (**C**) Differential metabolic pathways in *C. nasus* (95% confidence interval).

Both aquaculture-reared population (AP) and sedentary population (SP) *C. nasus* do not migrate; however, their food sources differ markedly, which influences the composition of their intestinal microflora. At the phylum level (Figure 6A), the dominant bacterial phyla in both groups are Firmicutes, Actinobacteriota, Proteobacteria, and Cyanobacteria, accounting for 86.16% to 90.84% of the total abundance. Notably, the relative abundance of Actinobacteriota is significantly higher in the AP *C. nasus* than in the SP *C. nasus* (35.05% vs. 8.37%). While at the genus level (Figure 6B), *Clostridium_T* was the dominant genus in both the AP and the SP *C. nasus*, accounting for 19.99% to 28.33% of the total abundance. Notably, the relative abundance of *Plesiomonas* was markedly higher in the SP *C. nasus* than in the AP *C. nasus* (10.28% vs. 0.02%).

Similarly, we performed a statistical analysis of the differential metabolic pathways between the AP and the SP of *C. nasus* (Figure 6C). The results showed that in the AP *C. nasus*, the enriched pathways included 4-hydroxyphenylacetate degradation and the superpathway of chorismate metabolism, whereas in the SP *C. nasus*, the enriched pathway was N10-formyl-tetrahydrofolate biosynthesis.

### 3.6. The Intestinal Microbiota of Migratory Freshwater and Sedentary Population C. nasus Differ Significantly

**Figure 7:**
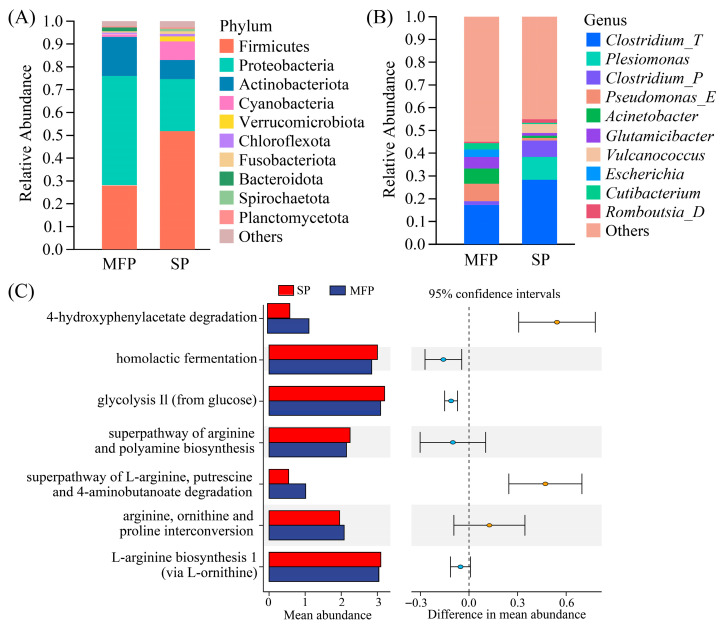
Statistical analysis of the intestinal microbiota composition and metabolic pathways in migratory freshwater populations and sedentary population *C. nasus*. MFP, Migratory Freshwater Population and SP, Sedentary Population. (**A**) Composition of dominant bacterial phyla in the intestine of *C. nasus* (relative abundance > 0.1%). (**B**) Composition of dominant bacterial genera in the intestine of *C. nasus* (relative abundance > 0.1%). (**C**) Differential metabolic pathways in *C. nasus* (95% confidence interval).

The migratory freshwater population (MFP) and the sedentary population (SP) *C. nasus* exhibit marked differences in life habits, reproductive behaviors, and physiological adaptations. Based on these distinctions, we aimed to characterize their intestinal microbiota. At the phylum level (Figure 7A), Proteobacteria, Firmicutes, and Actinobacteriota were the dominant phyla in both groups, accounting for 80.04% to 89.32% of the total abundance. Notably, the relative abundance of Cyanobacteria was substantially higher in the SP *C. nasus* than in the MFP (8.22% vs. 1.07%). At the genus level (Figure 7B), *Clostridium_T* was the dominant genus in both the MFP and the SP *C. nasus*, accounting for 17.13% to 28.33% of the total abundance. The relative abundances of *Pseudomonas_E* (7.83% vs. 1.29%), *Acinetobacter* (6.62% vs. 1.44%), and *Glutamicibacter* (4.95% vs. 0.68%) were significantly higher in the MFP *C. nasus* than in the SP ones. In contrast, the relative abundances of *Plesiomonas* (10.28% vs. 0.17%) and *Clostridium_P* (6.72% vs. 1.73%) were markedly higher in the SP *C. nasus* than in the MFP ones.

The metabolic pathways of the intestinal microbiota in the MFP and the SP *C. nasus* differed significantly (Figure 7C). In the MFP, the enriched pathways included 4-hydroxyphenylacetate degradation, the superpathway of L-arginine, putrescine, and 4-aminobutanoate degradation, as well as arginine, ornithine, and proline interconversion. By contrast, in the SP *C. nasus*, the enriched pathways were homolactic fermentation, glycolysis III (from glucose), and the superpathway of arginine and polyamine biosynthesis.

## 4. Discussion

### 4.1. Overview of the Intestinal Microbiota in C. nasus

The development of high-throughput sequencing technology has enabled comprehensive and efficient collection and analysis of intestinal microbiota data, thereby enhancing our understanding of the structure and function of the intestinal flora [34]. The primary objective of this research was to establish a more comprehensive, resolved catalog of bacterial taxa inhabiting the gut microbiota of *C. nasus*, and to characterize microbiome features linked to the integrated “life history–habitat–diet” model across its major ecotypes. A stratified sampling strategy was implemented to collect 280 individuals of *C. nasus* from 14 geographically distinct sampling sites, representing three ecotypes: migratory (comprising marine and freshwater subpopulations), sedentary, and aquaculture-reared. Intestinal luminal contents were then subjected to high-throughput 16S rRNA gene amplicon sequencing. A total of 37 phyla, 1144 genera, and 1707 species were identified in the sequencing results, demonstrating that the intestinal microbiota of *C. nasus* exhibits high diversity and occupies a relatively complex microbial ecological niche.

Species annotation revealed that Firmicutes, Proteobacteria, Actinobacteriota, and Cyanobacteria are the predominant bacterial phyla in the gut of *C. nasus*. Nie et al. employed PCR-DGGE fingerprinting technology to analyze the bacterial community structures in wild Yangtze River *C. nasus* and aquaculture-reared *C. nasus* [35,36]. Their results revealed that the dominant bacterial phyla in pre-migratory juveniles and post-migratory adults of wild *C. nasus* were Proteobacteria, Actinobacteria, and Firmicutes, whereas Proteobacteria and Actinobacteria predominated in aquaculture-reared *C. nasus*. This study significantly improved species resolution by leveraging a larger sample size and second-generation sequencing technology. It not only validated previous findings but also identified novel dominant phyla. Specifically, Firmicutes are involved in carbohydrate fermentation and produce short-chain fatty acids such as butyrate, which helps maintain intestinal barrier function and plays an essential role in fish health [37]. Proteobacteria contribute to maintaining the homeostasis of the intestinal anaerobic environment [38] and provide absorbable monosaccharide substrates for fish through the degradation of polysaccharides (e.g., cellulose and galactose) [39]. Actinobacteriota play a critical role in defending against pathogen invasion in *C. nasus* by producing antibiotics and secondary metabolites [40].

Similarly, the research results indicate that the dominant bacterial genera in *C. nasus* include *Clostridium_T*, *Plesiomonas*, *Clostridium_P*, and *Pseudomonas_E*. A comparison with previous studies reveals significant variations in the dominant bacterial genera among *C. nasus* of different ecological types. For example, Yang et al. identified *Rhodococcus* and *Photobacterium* as the predominant genera in the intestinal microbiota of *C. nasus* from the Yangtze River Estuary [28]. Duan et al. reported that the gut microbiota of *C. nasus* from Taihu Lake is primarily composed of *Bacteroides*, *Faecalibacterium*, *Halomonas*, and *Mycobacterium* [41]. Li et al. demonstrated that the gut microbiota of *Coilia nasus* from four water bodies—the mainstream of the Yangtze River (Pengze section), Poyang Lake, Qingcaosha Reservoir, and Shengsi Sea—is predominantly characterized by *Clostridium*, *Phocaeicola*, *Psychrobacter*, *Ralstonia*, *Acinetobacter*, and *Bacteroides* [42].

The composition of dominant bacterial genera exhibits significant variation across different ecological types of *C. nasus*. Mandal et al. demonstrated that a substantial proportion of microorganisms in fish are acquired from the water, food, and sediment in their growth environment [43]. Consequently, even within the same ecological type of *C. nasus*, intestinal microbiota can exhibit significant variation due to differences in developmental stage, diet, and collection basin. Specifically, members of *Clostridium* are frequently involved in polysaccharide degradation and short-chain fatty acid (SCFA) synthesis [44], whereas *Plesiomonas* can influence intestinal health by modulating the host immune response [45]. These findings suggest that the aforementioned bacterial genera may play a central role in the host’s nutritional metabolism.

Fish of different ecological types, having undergone long-term adaptation to distinct environmental conditions—such as salinity, temperature, diet, and migratory stress—exhibit significant differentiation in the diversity and structure of their intestinal microbiota. This divergence reflects the combined effects of host–microbe co-adaptation and environmental filtering [46,47,48]. The alpha diversity of the intestinal microbiota in the aquaculture-reared population (AP) and sedentary population (SP) of *C. nasus* is significantly higher than that in the anadromous (river–sea migratory) group. Furthermore, microbial variation among AP is the smallest, likely due to the relatively stable aquaculture environment and freshwater habitats (e.g., lakes), where food resources are more consistent and diverse. Such conditions provide a broader range of nutritional substrates and a stable niche for microbial colonization, thereby promoting the establishment and persistence of a more diverse gut microbiota. This observation is consistent with findings from Deng’s study on wild and farmed *Coreius guichenoti*, which similarly demonstrated higher microbial diversity in captive populations [49]. The Simpson diversity index shows minimal fluctuation, suggesting high community evenness and relative stability of dominant amplicon sequence variants (ASVs). Nevertheless, alpha diversity is generally dynamic across both short- and long-term temporal scales [50].

### 4.2. Salinity Significantly Influences the Composition of Intestinal Microbiota in Migratory Marine and Freshwater Populations of C. nasus

The salinity difference between seawater and freshwater is highly pronounced. The intestinal microbiota diversity in the migratory marine population (MMP) of *C. nasus* is lower compared to that in the freshwater population, potentially due to the inhibitory effects of high salinity on certain bacterial groups [27]. Elevated salinity imposes osmotic stress that inhibits the growth of freshwater and euryhaline microorganisms, thereby driving compositional shifts in microbial community structure [51]. Cui et al. discovered that the high salinity of seawater may suppress the growth of some obligate anaerobic bacteria (e.g., Bacteroides) [52], leading to slightly reduced bacterial diversity compared to freshwater fish.

In terms of species composition, *Photobacterium* and *Psychrobacter* were significantly enriched in the intestines of the MMP *C. nasus*. Notably, Photobacterium is a common luminescent bacterial genus found in marine fish [53]. Silva et al. previously identified *Photobacterium* spp. in the stomach and intestinal samples of *Lateolabrax japonicas* [54]. Its metabolic functions include salt ion regulation and lipid decomposition [55,56]. Fan et al. demonstrated that *Photobacterium* participates in the metabolism of carbohydrates and amino acids in the gut microbiota of *Hexagrammos otakii* [57]. The majority of *Psychrobacter* species have been isolated from various cold marine environments [58]. Studies indicate that Psychrobacter is a symbiotic member of the gastrointestinal microbiota in marine fish such as *Paralichthys adspersus*, *Oreochromis mossambicus*, and *Gadus morhua* [59,60,61]. Similarly, the relative abundance of *Clostridium_T* in the migratory freshwater population (MFP) of *C. nasus* was significantly higher than that in the MMP. Notably, common strains such as *Clostridium_sensu_stricto_1* dominate the intestines of juvenile *Acipenser dabryanus*, comprising over 70% of the anaerobic bacteria in their intestines [62]. This strain plays a critical role in promoting the production of short-chain fatty acids (SCFAs) and enhancing the structure of intestinal microbiota [63].

Moreover, the MMP of *C. nasus* exhibits enrichment of genes associated with aerobactin biosynthesis. Upon migrating into the sea, this population enters the fattening stage, during which feeding intensity markedly increases [64]. Consequently, the enrichment of this pathway may be associated with the elevated energy demands required for nearshore development in the migratory marine population of *C. nasus*. In contrast, pathways such as L-arginine degradation and 4-hydroxyphenylacetic acid degradation are enriched in the MFP of *C. nasus*, potentially reflecting its adaptation to fluctuating energy demands. During freshwater migration, *C. nasus* exhibits minimal feeding activity and relies on the conversion of non-sugar substances to sustain basic physiological functions [65].

### 4.3. The Dietary Composition Significantly Influences the Intestinal Microbiota Structure of Aquaculture-Reared and Sedentary Population C. nasus

Both aquaculture-reared population (AP) and sedentary population (SP) *C. nasus* populations are non-migratory and maintain stable food sources. However, a significant difference was observed in the dietary composition between the two groups. To ensure accelerated growth, the AP *C. nasus* are fed a combination of live prey—including cladocerans, copepods, and shrimp post-larvae—supplemented with nutritionally balanced artificial compound feed [66]. In contrast, the SP *C. nasus* primarily consume planktonic organisms like copepods and cladocerans, along with small aquatic animals [67]. The unique phylum Elusimicrobiota, detected in the SP *C. nasus*, is frequently observed in the intestines of ruminants [68]. Zhao et al. identified in their study on the intestinal microbiome of *Sus scrofa domesticus* that Elusimicrobiota plays a role in the synthesis of essential amino acids and vitamins [69]. Under aquaculture conditions, where diets are often monotonous, hosts may depend on microbial-assisted metabolism for nutrient acquisition [70]. The inclusion of plant fibers in artificial feed could potentially promote the colonization of this bacterial group. The abundance of *Plesiomonas* in the SP *C. nasus* is significantly elevated relative to other ecological communities. Prior evidence indicates that *Plesiomonas* (e.g., *P. shigelloides*) is detected in freshwater fish and may propagate indirectly through aquatic food webs [71]. Therefore, the elevated abundance of *Plesiomonas* in the intestines of sedentary *C. nasus* is tentatively associated with their zooplankton-dominated diet, while explicitly acknowledging that this inference lacks empirical support from direct dietary data.

The comparison of metabolic pathways reveals that the AP *C. nasus* exhibit enrichment in the 4-hydroxyphenylacetic acid degradation pathway, potentially linked to phenolic compounds present in artificial feed [72,73]. Phenolic substances promote digestive health and nutrient utilization by modulating the intestinal microbiota composition—specifically enriching beneficial bacterial populations—which enhances nutrient absorption and suppresses intestinal pathogens [74,75]. In contrast, the folate biosynthesis pathway enriched in the SP *C. nasus* may support their reproductive requirements under lower-nutrient conditions [76]. The pronounced differences in intestinal microbiota between the AP and the SP *C. nasus* underscore the significant impact of dietary sources.

### 4.4. Life History Strategies Shape the Intestinal Microbiota Communities of Migratory Freshwater and Sedentary Population C. nasus

Migratory *C. nasus* experience prolonged energy expenditure during migration and feed minimally during this period, whereas sedentary *C. nasus* exhibit lower movement intensity and have a more stable food supply. These differing life history strategies are the primary drivers of the distinct intestinal microbiota compositions observed in these two populations [77]. The number of bacterial phyla and genera in sedentary population (SP) *C. nasus* is significantly higher than that in migratory freshwater population (MFP) *C. nasus*. The unique phylum Campylobacterota found in the SP *C. nasus* correlates with specific organic matter conditions in the environment [78]. The enrichment of *Pseudomonas_E* in the MFP of *C. nasus* may enhance the host’s metabolic flexibility in variable environments. For instance, Ramasam et al. reported that different strains of *Pseudomonas* exhibit varying metabolic capabilities across diverse carbon sources [79]. The SP *C. nasus* inhabit lake ecosystems, where the high abundance of Cyanobacteria may be linked to the frequent occurrence of cyanobacterial blooms in their habitats (e.g., Taihu Lake and Chaohu Lake) [80]. This bacterial community can provide additional nitrogen sources for the host via nitrogen fixation [81].

The differences in metabolic pathways further substantiate the aforementioned hypothesis. The amino acid degradation pathway is potentially linked to energy metabolism in the MFP of *C. nasus* [65]. In contrast, glycolysis may serve as a key metabolic hub supporting the biosynthesis of secondary metabolites in the SP *C. nasus*, thereby augmenting its competitive fitness under stable environmental conditions [82]. The microbial distinctions between the anadromous freshwater population and the SP *C. nasus* underscore the influence of the host’s life history strategy on microbiota composition.

## 5. Conclusions

This study utilized high-throughput sequencing technology to analyze the compositional characteristics and metabolic functional differences in intestinal microbiota in four ecological groups of *C. nasus* (migratory marine, migratory freshwater, sedentary and aquaculture-reared populations), further elucidating the ecological adaptation mechanisms of host–microbiota interactions under varying life history strategies. A relatively comprehensive background database of *C. nasus* intestinal microbiota was established. Results demonstrated that the composition and function of *C. nasus* intestinal microbiota were significantly influenced by living environments and dietary sources, while migratory behavior also played a role in shaping distinct microbiota structures and metabolic features. The succession of metabolic functions in intestinal microbiota corroborated these findings.

These insights provide critical evidence for understanding the symbiotic mechanisms of fish–host–microbiota interactions. The research outcomes hold substantial significance for enhancing the background database of *C. nasus* intestinal microbiota and offer important implications for distinguishing *C. nasus* origins via intestinal microbiota analysis. Additionally, this study lays a foundation for future investigations into *C. nasus* microbiota ecology. However, PICRUSt2-based functional inference is inherently constrained by the phylogenetic breadth and functional completeness of its underlying reference genome database, introducing potential biases in predicted metabolic profiles. To address these limitations, predicted metabolic functions will be experimentally validated using shotgun metagenomic sequencing and untargeted metabolomics. Moreover, the precise mechanistic links between diet and the intestinal microbiota of *C. nasus* remain incompletely understood. Future studies should prioritize controlled comparative analyses of the gut microbiota in wild and farmed populations, integrating targeted dietary interventions to rigorously assess how specific nutritional components shape microbial community assembly, functional potential, and host-associated phenotypes. Such efforts hold translational promise for steering the microbiota of farmed *C. nasus* toward configurations characteristic of wild conspecifics, thereby supporting improved physiological homeostasis and environmental resilience.

## Figures and Tables

**Table 1:** Biological parameters of different ecological populations of *C. nasus*.

Type	Total Length (mm)	Body Length (mm)	Weight (g)	Supermaxilla to Head Length	Parasitic Number
Migratory Marine Population	346.05 ± 17.62	322.57 ± 16.52	143.39 ± 34.9	1.22 ± 0.07	6 ± 2
Migratory Freshwater Population	337.76 ± 23.00	311.09 ± 23.25	113.54 ± 25.44	1.17 ± 0.07	8 ± 7
Aquaculture-reared Population	225.19 ± 23.80	199.80 ± 17.06	25.72 ± 6.57	1.06 ± 0.04	0
Sedentary Population	248.24 ± 41.82	226.34 ± 40.28	51.01 ± 27.60	1.10 ± 0.06	0

Supermaxilla to head length (S/H): The length ratios of supermaxilla to head of *C. nasus*. The S/H ratio of *C. nasus* consistently exceeds 1.

## Data Availability

The raw sequence data reported in this paper have been deposited in the Genome Sequence Archive (Genomics, Proteomics & Bioinformatics 2021) in the National Genomics Data Center (Nucleic Acids Res 2022). China National Center for Bioinformation/Beijing Institute of Genomics, Chinese Academy of Sciences (GSA: CRA025833) that are publicly accessible at https://ngdc.cncb.ac.cn/gsa, accessed on 4 March 2026.

## Supplementary Materials

The following supporting information can be downloaded at https://www.mdpi.com/article/10.3390/ani16050840/s1. Supplementary Material S1. Figure S1. The results of otolith verification for *Coilia naus*. Figure S2. Rarefaction curve plot. Supplementary Material S2. Table S1. Dominant bacterial taxa in the intestinal microbiota of *Coilia nasus*. Table S2. Post-hoc test table of Alpha diversity index. Table S3. Beta diversity deffierence analysis. Table S4. Results of Venn diagrams.
