# Supplementary material for: Gut Microbiome Signatures Across Migratory, Sedentary, and Aquaculture Ecotypes of Coilia nasus
Source: Animals (Basel). 2026 Mar 7;16(5):840. doi: 10.3390/ani16050840 (PMC12984786; doi:10.3390/ani16050840)
Supplement: Supplementary file 1 [file animals-16-00840-s001.zip › Supplementary Material S1.pdf]

Figure S1.The results of otolith verification for *Coilia naus*

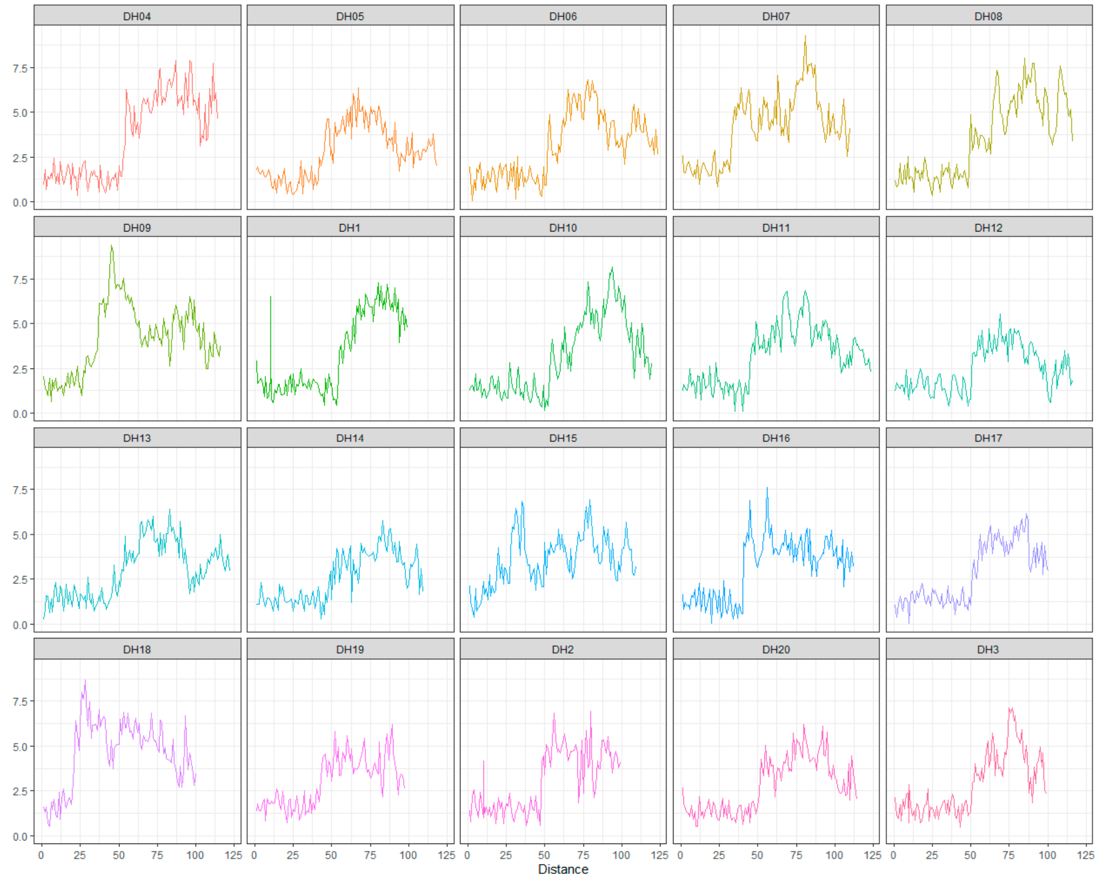

Figure legend: All individuals were confirmed as migratory *C. nasus* based on the Sr/Ca ratio analysis of oto-liths.

Figure S2. Rarefaction curve plot

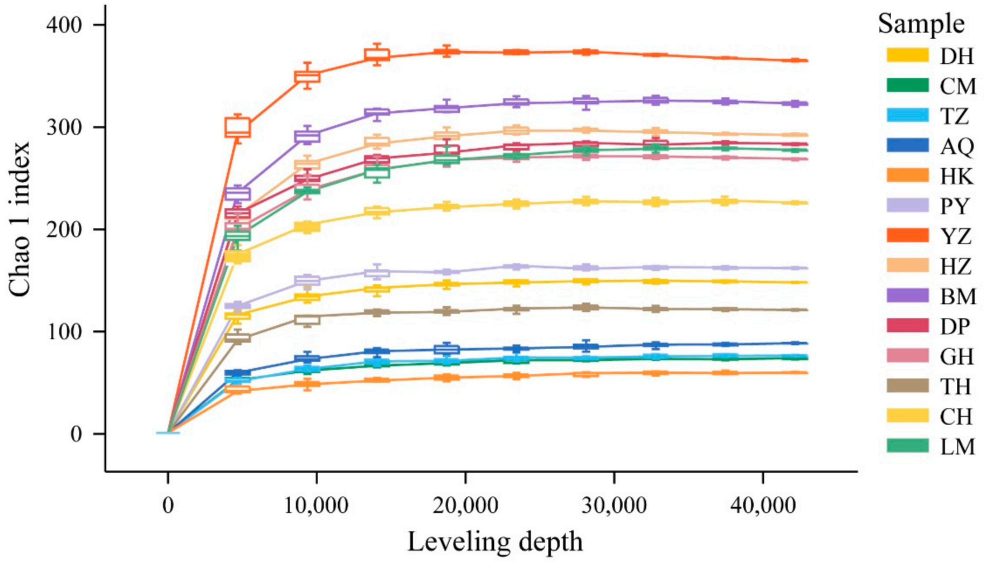

Figure S2. Rarefaction curve plot. The horizontal axis represents the rarefaction depth, and the vertical axis displays the median alpha diversity index (Chao1 index) across 10 independent rarefaction iterations, along with the corresponding box plot. In the box plot: the top and bottom edges of the box denote the third and first quartiles (i.e., the interquartile range, IQR); the central line indicates the median; and the upper and lower whiskers extend to the most extreme data points within 1.5 × IQR from the respective quartiles. The degree of curve flattening reflects how sensitive alpha diversity estimates are to sequencing depth.

Figure legend: A plateauing curve indicates that sampling depth is sufficient to capture the majority of microbial diversity present in the sample—further sequencing yields diminishing returns in terms of newly detected ASVs or OTUs. In contrast, a continuously rising curve suggests that alpha diversity remains undersampled and has not yet approached saturation.
